# *Arcobacter butzleri* is an opportunistic pathogen: recurrent bacteraemia in an immunocompromised patient without diarrhoea

**DOI:** 10.1099/acmi.0.000145

**Published:** 2020-06-12

**Authors:** Kerstin K. Soelberg, Trille K. L. Danielsen, Raquel Martin-Iguacel, Ulrik S. Justesen

**Affiliations:** ^1^​ Department of Clinical Microbiology, Odense University Hospital, Denmark; ^2^​ Department of Infectious Diseases, Odense University Hospital, Denmark

**Keywords:** *Arcobacter butzleri*, bacteraemia, immunosuppression, pathogen

## Abstract

**Introduction:**

*
Arcobacter butzleri* is attracting increasing interest due to its possible pathogenic properties. Researchers have described cases in which *A. butzleri* is isolated in stool samples from patients with gastrointestinal symptoms, mostly diarrhoea. The relevance of adding our case to the literature lies in its description of recurrent *A. butzleri* bacteraemia in a patient without diarrhoea.

**Case presentation:**

An immunocompromised patient was hospitalized three times within 12 months due to *A. butzleri-*induced bacteraemia. At no time did the patient experience diarrhoea even though examination of stool samples showed growth of *A. butzleri *. The isolate was susceptible to gentamicin, colistin and tetracyclines. The patient was successfully treated with doxycycline.

**Conclusion:**

For the first time in the literature we describe recurrent *A. butzleri* bacteraemia in a patient without diarrhoea. This case supports the classification of *A. butzleri* as an opportunistic pathogenic species, which clinical microbiology laboratories should be able to identify.

## Introduction

*Arcobacter butzleri* (formerly *Campylobacter butzleri *) is attracting increasing interest as an emerging pathogen associated with infectious diarrhoea [1]. A few case reports have described bacteraemia with *A. butzleri* [2–5], usually in connection with gastrointestinal symptoms. As the evidence in support of *A. butzleri* as a cause of infectious diarrhoea and status as a true pathogen remains ambiguous, the present case study represents an important addition to the literature. The potential pathogenicity of *A. butzleri* is described in connection with recurrent bacteraemia in a patient without diarrhoea, thus emphasizing the need for clinical microbiology laboratories to be able to identify this species.

## Case report

A 60–70-year-old patient in Denmark, with a known chronic autoimmune disease and kidney failure, was admitted to our hospital with fever, general malaise and erythema of the right lower extremity (Table 1). The patient had no diarrhoea. Cefuroxime (1.5 g×3) was administered intravenously. The next day *A. butzleri* was detected in two aerobic blood culture bottles. The patient was treated with intravenously administered ciprofloxacin (400 mg×2), with rapid treatment response. After 1 week the patient was discharged from the hospital. Based on susceptibility testing, treatment was changed to oral sulfamethoxazole-trimethoprim (MIC=0.5 mg l^−1^) for 7 days after discharge.

**Table 1.** Patient case information

Age	Comorbidity	Presenting features	Method of identification	Susceptibility data	Treatment	Outcome
Between 60 and 70 years old	A chronic autoimmune disease that causes inflammation in connective tissues, and kidney failure	First episode: fever (38.8 °C), general malaise, and erythema of the right lower extremity. C-reactive protein: 250 mg l^−1^. Leukocyte count: 12.7×10^9^ l^−1^.	MALDI TOF MS* and 16S rRNA gene sequencing	First episode: resistant to cefuroxime (no disc zone diameter) and ciprofloxacin (MIC=1 mg l^−1^) and susceptible to sulfamethoxazole-trimethoprim (MIC=0.5 mg l^−1^) and gentamicin (MIC=2 mg l^−1^).	First episode: cefuroxime, ciprofloxacin, sulfamethoxazole-trimethoprim	Cured
		Second episode: fever (40.1 °C), general malaise, and erythema of the left lower extremity. C-reactive protein: 314 mg l^−1^. Leukocyte count: 16.7×10^9^ l^−1^.		Second and third episodes: resistant to sulfamethoxazole-trimethoprim (MIC=8 mg l^−1^) and susceptible to gentamicin (MIC=2 mg l^−1^), colistin (MIC=0.25 mg l^−1^), and tigecycline (MIC=0.125 mg l^−1^).	Second episode: cefuroxime, gentamicin, doxycycline	
		Third episode: fever (38.7 °C) and general malaise. C-reactive protein: 113 mg l^−1^. Leukocyte count: 15×10^9^ l^−1^.			Third episode: doxycycline	

*MALDI-TOF MS, matrix-assisted laser-detected ionization-time of flight mass spectrometry.

Four months later the patient was readmitted with identical symptoms, this time with erythema of the left lower extremity. *A. butzleri* was detected in two aerobic blood culture bottles on the following day. The patient responded after initiation of cefuroxime. However, after susceptibility testing, gentamicin was added to the antibiotic treatment, as the isolate was found to be susceptible only to gentamicin (MIC=0.5 mg l^−1^), colistin (MIC=0.25 mg l^−1^) and tetracyclines (with tigecycline MIC=0.125 mg l^−1^ and 25-mm tetracycline disc zone diameter). Resistance to cefuroxime and sulfamethoxazole-trimethoprim (8 mg l^−1^) was found. Given the association between *A. butzleri* and diarrhoea*,* a stool sample was examined and growth of *A. butzleri* was detected after 1 day of incubation on 5 % horse blood agar in a normal atmosphere at 35 °C. 18F-fluorodeoxyglucose positron emission tomography/computed tomography imaging was performed, showing uptake in the skin of both legs and in the colon sigmoideum. Colonoscopy demonstrated diverticula along the entire colon, but without signs of diverticulitis. A transoesophageal echocardiography was also performed, indicating no signs of endocarditis. Treatment was changed to doxycycline for 10 days. The patient was discharged when a negative follow-up blood culture set was obtained.

Only 5 days after discharge, the patient felt unwell and was re-admitted; the sole symptoms now were fever and malaise. *A. butzleri* was detected in one aerobic blood culture bottle on the following day. Treatment with doxycycline was re-initiated, to which the patient responded quickly. The patient was discharged from the hospital after 7 days, with doxycycline treatment for another 5 weeks (6 weeks in total). When questioned about contact with farm animals and food routines, the patient reported no contact with animals and taking ready-made food deliveries from two different companies during the first and the second episode of bacteraemia. The significance of erythema of the lower extremities was never established, but response to antibiotic treatment was positive. The patient case information is summarized in Table 1.

We found excellent growth on 5 % horse blood agar after 1 day in all of the positive blood culture bottles and in the stool sample. The isolates were identified as *A. butzleri* by matrix-assisted laser-detected ionization time-of-flight mass spectrometry (MALDI-TOF MS; Bruker Biotyper), with a high confidence score. To confirm the diagnosis, 16S rRNA gene sequencing of the first 527 bp of the gene was performed after the second episode of bacteraemia using the MicroSeq 500 system (Perkin-Elmer, Applied Biosystems Division) as previously described [6]. Sequencing resulted in a definitive diagnosis of *A. butzleri* (467 bp sequence with a score of 99.57 % to the type strain RM4018). Sequencing results are available as supplementary material (available in the online version of this article). Susceptibility testing was performed with broth microdilution according to Riesenberg *et al*. with 5 % fetal bovine serum [7].

## Discussion

*A. butzleri* belongs to the genus *Arcobacter,* which are aerotolerant *Campylobacter *-like organisms [1]. Kiehlbauch *et al*. [8] originally described the species *Campylobacter butzleri* in 1991. It was assigned to the genus *Arcobacter* in 1992 [9]. Interest in *A. butzleri* has grown in recent years because of a surge in the number of cases describing an association between the finding of *A. butzleri* and gastrointestinal symptoms, with or without bacteraemia [1, 4]. *A. butzleri* is thought to be transmitted via contaminated food or water [5, 10]. A Danish study by Rasmussen *et al*. [10] showed that *A. butzleri* may be capable of persisting in a slaughterhouse environment, even after disinfection. *A. butzleri* has a number of genes that make it capable of environmental survival and tolerant of a wide range of atmospheres and temperatures [11, 12].

The complete genome sequence and analysis of *A. butzleri* from a human clinical strain was performed in 2007 [11]. Putative virulence determinants were found, some of which were identical to *Campylobacter jejuni *, thus supporting the potential pathogenicity of *A. butzleri *. However, it has been speculated that only specific strains, or the status of the host, determine the pathogenicity of *A. butzleri *, as no difference was found in the prevalence of *A. butzleri* in stool samples from cohorts with and without diarrhoea [12].

Unrecognized cases of *A. butzleri* diarrhoea or bacteraemia are likely to occur if MALDI-TOF MS is not available and because laboratories typically do not look for *A. butzleri* in stool samples. Phenotypic identification is very difficult, and there is no evidence to indicate that a single medium, temperature or atmosphere will isolate all strains of *A. butzleri* [12]. Even with MALDI-TOF MS, it has been reported that enriched databases are needed to improve sensitivity [13].

We present the first case of recurrent *A. butzleri* bacteraemia without any specific gastrointestinal symptoms. Despite the absence of diarrhoea we were able to isolate *A. butzleri* from stool samples, leading us to assume that the bacteraemia originated from the colon. We do not know whether the three cases of bacteraemia were caused by the same strain of *A. butzleri *. The first and the second episodes were 4 months apart and may have been caused by two different strains. However, the second and third cases of bacteraemia were most likely to be caused by the same strain, with the third case a result of treatment failure caused by the short duration of therapy.

We identified four previously published case reports [2–5] of *A. butzleri* bacteraemia (Table 2). With the addition of our case, four out of the five known cases [2, 3, 5] involved immunocompromised patients. This would support the conjecture of Webb *et al*. [12] that *A. butzleri* is an opportunistic pathogen.

**Table 2.** Published case reports with *Arcobacter butzleri* bacteraemia

Case report	Sex/age	Comorbidity	Presenting features	Method of identification	Susceptibility data	Treatment	Outcome
On *et al*. [2] (1995)	M/1 day	Preterm labour	Hypotension, hypothermia and hypoglycaemia	Phenotypic	Resistance to amoxicillin, piperacillin, cefuroxime, ceftazidime, cefotaxime, amoxicillin-clavulanic acid and trimethoprim	Penicillin, cefotaxime	Cured
Yan *et al*. [3] (2000)	M/60 years	Chronic hepatitis B, liver cirrhosis	Fever (39.5 °C), haematemesis, distended abdomen, and pitting oedema in the lower extremities. Leukocyte count: 12.0×10^9^ l^−1^.	16S rRNA gene sequencing	Ampicillin 24 mg ml^−1^; amoxicillin-clavulanic acid 6.0/3.0 mg ml^−1^; cephalothin >256 mg ml^−1^; cefuroxime 96 mg ml^−1^; cefotaxime 24 mg ml^−1^; and clarithromycin 3.0 mg ml^−1^	Cefuroxime	Cured
Lau *et al*. [4] (2002)	F/69 years	None	Fever (38.0 °C), and right lower quadrant pain. Leukocyte count: 18.1×10^9^ l^−1^.	16S rRNA gene sequencing	Resistance to cephalothin and susceptible to nalidixic acid	Cefuroxime, metronidazole	Cured
Arguello *et al*. [5] (2015)	M/85	Chronic lymphocytic leukaemia	Fever (39.3 °C), hypotension, diffuse maculopapular rash on the skin, serous wounds on the right lower extremity, and diarrhoea. Leukocyte count: 15.2×10^9^ l^−1^.	16S rRNA gene sequencing	Unable to perform susceptibility testing	Vancomycin, piperacillin-tazobactam	Cured

This case supports the finding that *A. butzleri* is rightfully classified as an opportunistic pathogen, although further research into host factors and specific *A. butzleri* strain factors may qualify this assumption. However, we recommend that clinical microbiology laboratories are equipped to identify this species.

## Supplementary Data

Supplementary material 1
