# Supplementary material for: Arcobacter butzleri is an opportunistic pathogen: recurrent bacteraemia in an immunocompromised patient without diarrhoea
Source: Access Microbiol. 2020 Jun 12;2(8):acmi000145. doi: 10.1099/acmi.0.000145 (PMC7497825; doi:10.1099/acmi.0.000145)
Supplement: Supplementary material 1 [file acmi-2-145-s001.pdf]

Arcobacter butzleri RM4018, complete genome, Sequence ID: CP000361.1

Alignment statistics for match with case isolate (second episode)				
Score	Expect	Identities	Gaps	Strand
852 bits(461)	0.0	465/467(99.57%)	0/467(0%)	Plus/Plus

```
Query  1       GCTCAGAGTGAACGCTGGCGGCGTGCTTAACACATGCAAGTCGAACGAGAACGGATTATA  60
               ||||||||||||||||||||||||||||||||||||||||||||||||||||||||||||
Sbjct  847479  GCTCAGAGTGAACGCTGGCGGCGTGCTTAACACATGCAAGTCGAACGAGAACGGATTATA  847538

Query  61      GCTTGCTATAATTGTCAGCTAAGTGGCGCACGGGTGAGTAATGTATAGGTAATATGCCTC  120
               ||||||||||||||||||||||||||||||||||||||||||||||||||||||||||||
Sbjct  847539  GCTTGCTATAATTGTCAGCTAAGTGGCGCACGGGTGAGTAATGTATAGGTAATATGCCTC  847598

Query  121     TTACTAAGGGATAACAATTGGAAACGATTGCTAATACCTTATATTCCTTTTTATCAAAAG  180
               ||||||||||||||||||||||||||||||||||||||||||||||||||||||||||||
Sbjct  847599  TTACTAAGGGATAACAATTGGAAACGATTGCTAATACCTTATATTCCTTTTTATCAAAAG  847658

Query  181     ATAAAAAGGGAAAGATTTATTGGTAAGAGATTAGCCTGTATTGTATCAGTTAGTTGGTGG  240
               ||||||||||||||||||||||||||||||||||||||||||||||||||||||||||||
Sbjct  847659  ATAAAAAGGGAAAGATTTATTGGTAAGAGATTAGCCTGTATTGTATCAGTTAGTTGGTGG  847718

Query  241     GGTAATGGCCTACCAAGACGATGACGCATAACTGGTTTGAGAGGATGATCAGTCACACTG  300
               ||||||||||||||||||||||||||||||||||||||||||||||||||||||||||||
Sbjct  847719  GGTAATGGCCTACCAAGACGATGACGCATAACTGGTTTGAGAGGATGATCAGTCACACTG  847778

Query  301     GAACTGAGACACGGTCCAGACTCCTACGGGAGGCAGCAGTGGGGAATATTGCACAATGGA  360
               ||||||||||||||||||||||||||||||||||||||||||||||||||||||||||||
Sbjct  847779  GAACTGAGACACGGTCCAGACTCCTACGGGAGGCAGCAGTGGGGAATATTGCACAATGGA  847838

Query  361     CGAAAGTCTGATGCAGCAACGCCGCGTGGAGGATGACACATTTCGGTGCGTAAACTCCTT  420
               ||||||||||||||||||||||||||||||||||||||||||||||||||||||||||||
Sbjct  847839  CGAAAGTCTGATGCAGCAACGCCGCGTGGAGGATGACACATTTCGGTGCGTAAACTCCTT  847898

Query  421     TTATATAAGAAGATAATGACGGTATTATATAATTAAGCACCGGCTAA  467
               |||||||||||||||||||||||||||||| | ||||||||||||||
Sbjct  847899  TTATATAAGAAGATAATGACGGTATTATATGAATAAGCACCGGCTAA  847945
```
